# Sleep Quality's Effect on Vigilance and Perceptual Ability in Adolescent and Adult Athletes

**DOI:** 10.1155/2021/5585573

**Published:** 2021-04-11

**Authors:** Vasileios T. Stavrou, Kyriaki Astara, Konstantinos N. Tourlakopoulos, Zoe Daniil, Konstantinos I. Gourgoulianis, Konstantinos Kalabakas, Dimitrios Karagiannis, George Basdekis

**Affiliations:** ^1^Laboratory of Cardio-Pulmonary Testing and Pulmonary Rehabilitation, Department of Respiratory Medicine, Faculty of Medicine, University of Thessaly, Larissa, Greece; ^2^The Medical Project, Prevention, Evaluation and Recovery Center, Larissa, Greece

## Abstract

The aim of the study was to investigate the effect of sleep quality in cognitive domains of perceptual ability after exhausting exercise in adolescent and adult athletes. Eighty-six male professional soccer players were included in our study and divided into two groups: adolescents (age: 17.3 ± 0.2 yrs, body mass: 68.9 ± 7.9 kg, body fat: 9.9 ± 3.6 %) versus adults (age: 26.3 ± 5.2 yrs, body mass: 76.5 ± 7.2 kg, body fat: 10.3 ± 3.1 %). For each athlete, prior to cardiopulmonary exercise testing (CPET), anthropometric and morphological characteristics were recorded and Pittsburgh Sleep Quality Index (PSQI) questionnaire was answered. Immediately after CPET, all athletes underwent the perceptual ability test (PATest) for 30 sec and the sum of hits (rep/30 sec) and the time between a visual stimulus and the following stimulus (mean reaction time; RT, sec) were recorded. Oxygen uptake in maximal effort and in anaerobic threshold showed differences between hits (*P*=0.037) and RT (*P*=0.025). The variable of PSQI questionnaire “*had bad dreams*” showed correlation with hits (*P*=0.021) and RT (*P*=0.011) and the RT showed correlation with variables “cannot breathe comfortably” (*P*=0.041) and “*...enthusiasm to get things done*” (*P*=0.041). Adolescents showed poorer sleep quality (PSQI score: 5.7 ± 3.6 vs. 2.4 ± 2.6) compared to adults and slower reaction time (0.9 ± 0.1 vs. 0.8 ± 0.1 sec, *P*=0.029) compared to adolescent athletes with PSQI score ≥5.5. The variable of PSQI score in adolescents is related to HR in maximal effort (*r* = −0.364, *P*=0.032) and in adults is related to speed (*r* = −0.335, *P*=0.016). Perceptual ability, which requires sustained attention, vigilance, and motor coordination, is often negatively affected by restricted sleep, especially in adolescents.

## 1. Introduction

Soccer is one of the most popular team sports practiced around the world. During the season, elite male soccer players practice on a daily basis, often twice a day, play one or two matches per week, and take part in national and/or international tournaments [1]. This schedule requires well developed physical, mental, and physiological characteristics [1] and optimal recovery to reduce the risk of transitioning into a state of excessive fatigue as well as to reduce the risk of injury [2].

Sleep quality, in high level athletes, is affected by the kind of sport, the type of exercise, the training frequency, psychobiological mechanisms, and chronotype [3]. Adequate sleep is essential for peak performance while sleep deprivation (SD) has been shown to negatively affect many physiological, cognitive, and behavioral measures [4] and associated with longer reaction times and reduced force on a simple and choice reaction time test (Baseline: 244 ± 39 versus SD: 281 ± 31 ms) [5]. Moreover, sleep deprivation reduces the fitness indicators (such as *V*O_2max_) without affecting anaerobic power as recording after Wingate test [6]. Athletes with poor sleep quality and quantity have higher levels of confusion compared to athletes reporting better sleep, while the athletes who usually lose in team sports have higher levels of tension and confusion before the game [7]. Enhancing sleep quality and quantity is a fundamental tool to promote multidimensional recovery for athletes, in the context of maintaining physical condition and alertness [2]. However, alertness is only one of the several cognitive functions that are all neurally interlinked and thus hindered by sleep deprivation [8]. Perceptual functions, as a whole, have not been adequately studied in relation to exercise and sleep characteristics. To this end, we designed this study to investigate the effect of sleep quality in several cognitive components of perceptual ability after exhausting exercise between adolescents and adults. We hypothesized that quality and quantity of sleep could affect the fitness indicators and parameters of perceptual ability.

## 2. Methods

### 2.1. Participants

Eighty-six Greek male professional soccer players (age, 22.7 ± 6.0 yrs, training age, 9.3 ± 4.9 yrs, body fat, 10.1 ± 3.3%, Table 1), from the Greek Super League 1 and 2, were included in our study on a voluntary basis, during preparation period between July 2019 and August 2019 and two months before the official games started. All athletes were divided into two groups: adolescents (≤18 years old) versus adults (>18 years old) Table 1. Inclusion criteria were age between <15 and >35 years old, training age ≥4 years (≥600 min per week with HR ≥70% of max), and not having a recent injury (for the last 12 months) [9]. All volunteers have lived and been trained in less than 100 m altitude at sea level, for above 10 months [10]. The study was conducted according to the Helsinki declaration for use in Human subjects (N° 58076/14-11-2018, Scientific Council of University Hospital of Larissa, Greece) and personal data [137/Α/29-8-2019 and EC 2016/679] according to European Parliament and of the Council of the European Union. All the participants, coaches, team chiefs, and parents submitted a written consent.

### 2.2. Procedures

For each athlete, prior to cardiopulmonary exercise testing (CPET), anthropometric and morphological characteristics (Table 1) and body composition (Tanita MC-980, Illinois, USA) were recorded. All athletes answered Pittsburgh Sleep Quality Index (PSQI) questionnaire [9, 11] and it was recorded in their medical history.

CPET was performed on an electronic treadmill ergometer (Cosmos LE 100CE-720CE, Germany). For each athlete, O_2_ uptake (FitmateMED Cosmed, Italy) and heart rate (Garmin, Kansas, USA) were recorded during CPET (Table 1). Prior to testing, all athletes were familiarized with the test via a 2 min^−1^, in resting and upright position (1^st^ stage). In 2nd stage, all athletes started the test with speed 5 km/h^−1^ and increment 1 km per min^−1^, until exhausting. After the end of the maximal effort (2^nd^ stage), they performed a 5 min^−1^ walking (3^rd^ stage) for recovery purposes, with speed 3 km/h^−1^ [12]. The slope of the treadmill during CPET was steadily 1%.

**Figure 1:**
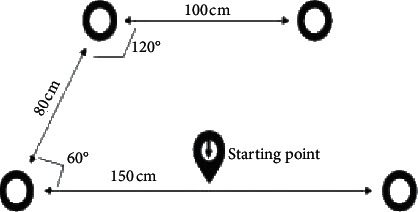
Dimensions of Fitlight Trainer^®^ during PATest.

Immediately after CPET, all athletes underwent the perceptual ability test (PATest) to evaluate the accuracy to visual stimulus by Fitlight Trainer® test. The Fitlight Trainer® (FitLight Sports Corp, Canada; available in Greece by Serinth®) is a wireless reaction system comprised of four LED lights controlled by a tablet computer. The lights have an inbuilt sensor which reacts proximity or touch and deactivates the light [13]. Four lights positioned on the floor were used as stimulus (Figure 1) and turning off the lights was done with feet. The frequency between signals was 2 sec. All athletes did one trial for 30 sec and recorded the sum of hits (rep/30 sec) and the time interval between a visual stimulus and the following stimulus (mean reaction time; RT, sec). None of the participants stated they had previous experience in this test, so learning effect does not exist.

All sessions were performed in The Medical Project Center, Larissa, Greece, with environmental temperature at 22 ± 1°C and humidity 45 ± 3%. The evaluation was made between 10:00 a.m. and 15:00 p.m. and all athletes did not have a previous exercise and/or training for 48 hours.

### 2.3. Statistical

Kolmogorov–Smirnov test was utilized to assess normality of distribution of values. Spearman's Rho and Pearson's R correlation coefficients were used to determine correlations between continuous variables where appropriate. Independent samples *t*-tests and Mann–Whitney *U*-test were used to compare rates of change for adolescents (≤18 years old) and adults (>18 years old) and cross-sectional point in PSQI score 5.5 was used to separate good versus bad sleepers [3]. Regression analysis was performed for multivariate adjustments for all covariates, simultaneously. For all aforementioned tests, a *P* value <0.05 was considered statistically significant and the data are presented as mean value, standard deviation (mean ± SD), and 95% confidence intervals. The SPSS 25 statistical package (SPSS Inc., Chicago, Illinois, USA) was used for the statistical analyses.

## 3. Results

### 3.1. Cardiopulmonary Exercise Testing

Results showed differences between fitness indicators and PATest (Table 1). Oxygen uptake in maximal effort (*V*O_2max_, 58.1 ± 4.9 ml/min^−1^/kg^−1^) showed statistical significance with Hits (*r* = −0.279, *P*=0.037) and RT (*r* = −0.365, *P*=0.025) and oxygen uptake in anaerobic threshold (45.2 ± 4.2 ml/min^−1^/kg^−1^) showed statistical significance with Hits (*r* = 0.397, *P*=0.042) and RT (*r* = 0.389, *P*=0.026). Moreover, *V*O_2max_ showed statistical significance with PSQI parameter “*cannot breathe comfortably*” (*r* = 0.241, *P*=0.025) and *V*O_2_ in anaerobic threshold, as a percent of maximal uptake (*V*O_2anaerobic threshold_: 77.8 ± 5.5% of max), with parameters of PSQI “cannot get to sleep within 30 minutes” (*r* = 0.281, *P*=0.009), “*cough or snore loudly*” (*r* = 0.293, *P*=0.006), and PSQI score (*r* = 0.254, *P*=0.019). Adolescents showed lower values in parameters of *V*O_2anaerobic threshold_ (46.4 ± 4.6 vs. 44.3 ± 3.7 ml/min^−1^/kg^−1^, 95% CI: 0.321 to 3.902, *t*_(84)_ = 2.345, *P*=0.021), *V*O_2max_ (59.7 ± 6.2 vs. 57.0 ± 3.6 ml/min^−1^/kg^−1^ 95% CI: 0.618 to 4.801, *t*_(84)_ = 2.576, *P*=0.012) and higher values in HR in maximal effort (197.9 ± 8.3 vs. 191.1 ± 10.3 bpm, 95% CI: 2.673 to 11.055, *t*_(84)_ = 3.257, *P*=0.002) compared to adults. The variable of PSQI score in adolescents was related to HR in maximal effort (*r* = −0.364, *P*=0.032) and in adults was related to duration (*r* = −0.352, *P*=0.011) and speed (*r* = −0.335, *P*=0.016).

### 3.2. Sleep Quality

**Figure 2:**
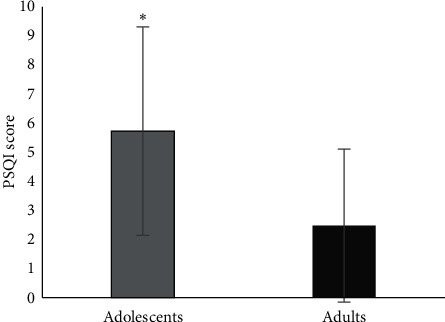
Relationship between adolescents and adults soccer players in PSQI score. *∗P* < 0.001.

Results showed differences between age groups and PSQI variables (Table 2). Adolescents showed poorer sleep quality compared to adults (Figure 2). More specifically, adolescents scored higher in variabilities “*wake up in the middle of the night or early morning”* (1.5 ± 1.1 vs. 0.6 ± 0.9, 95% CI: 0.378 to 1.242, *t*_(84)_ = 3.728, *P* < 0.001), “*have to get up to use the bathroom*” (1.1 ± 0.9 vs. 0.6 ± 0.8, 95% CI: 0.153 to 0.938, *t*_(84)_ = 2.762, *P*=0.007), “*cannot breathe comfortably*” (0.1 ± 0.4 vs. 0.1 ± 0.1, 95% CI: 0.002 to 0.226, *t*_(84)_ = 2.042, *P*=0.046), “*cough or snore loudly*” (0.3 ± 0.7 vs. 0.1 ± 0.2, 95% CI: 0.041 to 0.470, *t*_(84)_ = 2.364, *P*=0.020), “*feel too hot*” (1.1 ± 1.0 vs. 0.4 ± 0.8, 95% CI: 0.309 to 1.153, *t*_(84)_ = 3.442, *P*=0.001), “*have bad dreams*” (0.4 ± 0.7 vs. 0.1 ± 0.4, 95% CI: 0.074 to 0.566, *t*_(84)_ = 2.584, *P*=0.012), “*…how often have you had trouble staying awake while….*” (0.3 ± 0.6 vs. 0.1 ± 0.2, 95% CI: 0.023 to 0.373, *t*_(84)_ = 2.253, *P*=0.027), and “*…enthusiasm to get things done*” (0.4 ± 0.6 vs. 0.1 ± 0.4, 95% CI: 0.075 to 0.507, *t*_(84)_ = 2.679, *P*=0.009) compared to adults. Adolescents showed correlation with reaction time in PSQI variables “*cannot breathe comfortably*” (*r* = 0.410, *P*=0.014) and “*have bad dreams*” (*r* = 0.357, *P*=0.035). The athletes with PSQI score ≥5.5 were younger (19.3 ± 4.0 vs. 24.3 ± 6.2 yrs, 95% CI: −7.523 to −2.386, *t*_(84)_ = −3.836, *P* < 0.001) and had lower training age (7.6 ± 3.7 vs. 10.1 ± 5.3 yrs, 95% CI: −4.618 to −0.199, *t*_(84)_ = −2.169, *P*=0.033). Moreover, athletes with PSQI score ≥5.5 showed lower reduction of HR at the first minute of recovery stage (28.2 ± 11.7 vs. 35.6 ± 9.9 bpm, 95% CI: 3.239 to 12.836, *t*_(84)_ = 2.867, *P*=0.005) and lower reaction time (0.9 ± 0.2 vs. 0.8 ± 0.9 sec, 95% CI: 0.008 to 0.137, *t*_(84)_ = 2.186 *P*=0.036), compared to athletes with PSQI score <5.5. In addition, the adolescent athletes with PSQI score ≥5.5 displayed slower reaction time (0.9 ± 0.1 vs. 0.8 ± 0.1 sec, 95% CI: 0.011 to 0.203, *t*_(33)_ = 2.277, *P*=0.029) compared to adolescent athletes with PSQI score <5.5. The adult athletes did not show significant differences by cross-sectional point in PSQI score 5.5.

### 3.3. Perceptual Ability Test

**Figure 3:**
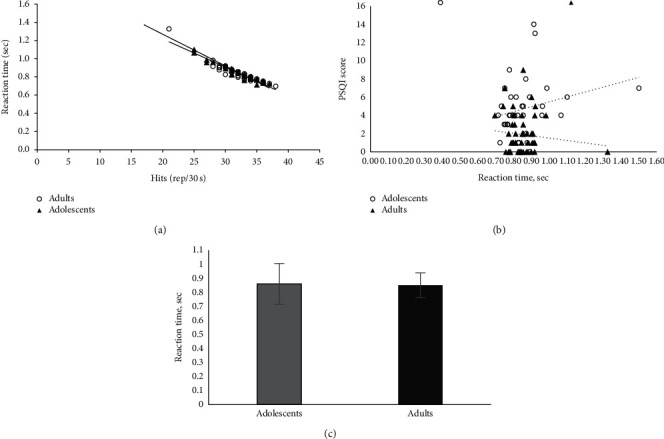
Relationship between reaction time and (a) hits (adults: *y* = −0.031*x* + 1.8381, *R*^2^ = 0.8726, *P* < 0.001; adolescents: *y* = −0.0348*x* + 1.9653, *R*^2^ = 0.9362, *P* < 0.001), (b) PSQI score (adults: *y* = −2.6294*x* + 4.1389, *R*^2^ = 0.0126, *P*=0.265; adolescents: *y* = 5.3768*x* + 0.1151, *R*^2^ = 0.1663, *P*=0.046), and (c) age group.

Results showed a difference between variabilities during perceptual ability test (reaction time: 0.855 ± 0.114 s, Hits: 31.8 ± 3.2 rep/30 s). Athletes with lower reaction time showed higher count of hits on deactivating the light (Fitlight Trainer®), compared to athletes with higher score on reaction time (Figure 3(a)) and athletes with poorer sleep quality had lower reaction time (Figure 3(b)). Moreover, the reaction time did not show significant differences between adolescents and adults (Figure 3(c)).

### 3.4. Athletes' Characteristics

Results showed differences between age groups and characteristics (Table 1). For the total of athletes, the variable muscle mass showed correlation with PSQI questionnaire variables “*cannot get to sleep within 30 minutes*” (*r* = −0.268, *P*=0.013), “*feel too hot*” (*r* = −0.329, *P*=0.002), and “*had bad dreams*” (*r* = −0.320, *P*=0.003). The age showed correlation with PSQI questionnaire variables “*cannot get to sleep within 30 minutes*” (*r* = −0.272, *P*=0.011), “*wake up in the middle of the night*” (*r* = −0.325, *P*=0.002), “*cough or snore loudly”* (*r* = −0.236, *P*=0.029), “*feel too hot*” (*r* = −0.361, *P*=0.001), and “*had bad dreams*” (*r* = −0.281, *P*=0.009). Adolescents showed lower values in parameters of body composition variabilities (body mass: 68.9 ± 7.9 vs. 76.5 ± 7.2 kg, 95% CI: −10.877 to −4.337, *t*_(84)_ = −4.426, *P* < 0.001; body mass index: 21.9 ± 1.8 vs. 23.5 ± 1.3 kg/m^2^, 95% CI: −2.239 to −0.879, *t*_(84)_ = −4.558, *P* < 0.001; muscle mass: 57.9 ± 7.1 vs. 65.2 ± 5.7 kg, 95% CI: −10.111 to −4.587, *t*_(84)_ = −5.292, *P* < 0.001; body surface area: 1.7 ± 0.2 vs. 1.9 ± 0.2 m^2^, 95% CI: −0.326 to −0.115, *t*_(84)_ = −4.145, *P* < 0.001 and lean body mass: 56.2 ± 4.6 vs. 60.1 ± 4.5 %, 95% CI: −5.947 to −2.010, *t*_(84)_ = −4.020, *P* < 0.001, Table 1) compared to adults (Table 1). The variable of age in adolescent group showed correlation with PSQI score (*r* = −.418, *P*=0.013). The variable muscle mass in adolescent group showed correlation with PSQI questionnaire variable “*cannot get to sleep within 30 minutes*” (*r* = −0.371, *P*=0.028). The variable of PSQI questionnaire “*feel too hot*” showed correlation, in adult group, with variable of body surface area (*r* = −0.283, *P*=0.044) and lean body mass (*r* = −0.281, *P*=0.046).

Multiple R of regression analysis was .91 which is statistical difference to zero, *F*_(21, 64)_ = 13.771, *P* < 0.001. A total of 76% of the age of athletes was interpreted through variabilities: training age (*t* = 8.96, *P* < 0.001), total body water (*t* = −2.18, *P*=0.033), muscle mass (*t* = 2.66, *P*=0.010), HR in maximal effort (*t* = −2.45, *P*=0.017), and PSQI score (*t* = −2.627, *P*=0.011).

## 4. Discussion

The data from the present study reveal an interrelationship among sleep quality, perceptual ability, and performance, which is more pronounced in adolescents. Sleep is a key component in an athlete's recovery process [9]. Sleep deprivation reflects symptoms of overtraining, both physical and mental, in periods of increasing training load, stress, and muscle and bone injuries [14]. Chronic and/or acute sleep loss is directly correlated with athletic injuries and/or “*fatigue-related injuries*” and this reduced amount of sleep is a direct, independent risk factor for injuries during exercise, diminishing the physical aspect of athletic performance [15].

### 4.1. Cardiopulmonary Fitness

Our results indicated a relationship among *V*O_2max_ and PSQI parameter “*cannot breathe comfortably*” and *V*O_2_ at anaerobic threshold (as a percent of *V*O_2max_), with parameters of PSQI “*cannot get to sleep within 30 minutes,*” “*cough or snore loudly,*” and PSQI score. These could be partly attributed to period and kind of training. The aerobic and anaerobic capacity are related to different competitive levels, playing positions, age groups, and interactions among these factors [1], while sleep deprivation is related to various factors that lower *V*O_2max_, such as reduced submaximal strength [16]. In the present study, higher scores in variabilities of PSQI questionnaire were observed in athletes with lower *V*O_2_ in anaerobic threshold, as a percent of maximal uptake, compared to athletes with higher *V*O_2_ in anaerobic threshold. In agreement with our findings, Vanuxem et al. [17] reported sleep deprivation relate to muscle metabolism with elevated levels of lactic acid concentration in the blood and decreased ability to remove it during exercise. In addition, Taheri and Arabameri [6] reported that anaerobic power is not affected following one-night sleep deprivation.

Our data present that athletes were classified as good sleepers at the total PSQI score (3.1 ± 2.9) compared to previous studies where athletes were classified as poor sleepers when having score >5.5 in PSQI [3]. The PSQI is a questionnaire subjected to the participant's self-assessment of sleep quality [11] during the last 30 days and incorporates both qualitative and quantitative aspects. The athletes enrolled were recruited during their preparation period. Although the participants' subjective rate of overall sleep quality is satisfying (3.1 ± 2.9), PSQI scores display particular sleep disturbances between the reaction time and PSQI parameters such as “*cannot breathe comfortably,*” “*have bad dreams,*” and “*…enthusiasm to get things done.*” Difficulties in breathing during sleep, such as PSQI parameter “*cannot breathe comfortably*”, have been classified as sleep-related breathing disorders (SDB), which comprise a group of disorders from habitual snoring to obstructive sleep apnea (OSAS). Symptoms range from snore to periodic episodes of apnea in terms of breathing. Interestingly, the most prevalent manifestation of SDB is not disordered breathing like snoring, but excessive sleepiness, indicating the correlation between sleep breathing and cognition [18]. Hence, snoring is not innocent at all, for it is capable of initiating a pathophysiological cascade from events, like intermittent hypoxia, to diminished sleep quality [19, 20] and athletic performance.

According to Yoshitaka et al. [21], teenage athletes with SDB had higher heart rate in sleep compared to non-SDB athletes. SBD in athletes, with no clinical manifestations developed, could disturb the balance between the sympathetic and parasympathetic activity in ways that potentially may lead to adverse cardiovascular complications [22]. In the present study, it was shown that athletes with PSQI score >5.5 displayed slower recovery of HR during the first minutes of recovery stage compared to athletes with lower score (PSQI score <5.5). This chronotropic incompetence at recovery stage during exercise reflects the sympathetic and parasympathetic activity. In agreement with our previous findings [12], sleep deprivation results in instability of the autonomic nervous system, which is associated with endothelial dysfunction and sympathetic induced vasoconstriction. The main causative factor, for chronotropic incompetence, was proposed to be impaired cardiovascular autonomic function resulting from structural down regulation of cardiac *β*-receptors and/or altered baroreflex set-point. Relevant studies by Costa et al. [23] enhanced this notion by providing evidence about athletes' chronobiology and environmental aspects in association with sleep and autonomic features.

### 4.2. Perceptual Ability Test

The correlation between variables of PSQI and PAT indicates of a broader association between quality and patterns of sleep and cognitive performance during exercise. Decreased performance and recovery for athletes [24] and deficits in attention and vigilance [25] have been attributed to poor sleep. During rapid eye movement sleep (REM) sleep, apneic episodes are exacerbated [26], making its complications, including restless dreaming, more prominent. Dreams are most common in REM [27], in which they tend to be more vivid, bizarre, and aggressive, as well as more memorable [28]. The PSQI parameter “*have bad dreams*” constitutes an additional indicator of poor quality of sleep, correlated with decreased perceptual ability. Since participants claim to have bad dreams, it is possible that they are awakened during REM sleep, resulting in sleep fragmentation and deprivation. Although it cannot be determined in the present study whether sleep deprivation is total or spatial, cognitive performance is adversely affected in both conditions, in terms of diminished attention span, decision-making ability, and reaction time [29].

Furthermore, athletes report less “*...enthusiasm to get things done,*” which correlated with less reaction time in PATest. A low motivation in, e.g., training, perhaps due to daytime somnolence or not optimal recovery, indicates the necessity to reduce energy expenditure, as the stressful stimuli are inversely associated with physical activity [30]. This further undermines the preservation of muscle mass because of the reduction in training work load itself. Another candidate etiologic model for lack of motivation observed comprises the affected vigilance and motor coordination, as further neurocognitive complications of SDB [31]. Impaired attention/vigilance could either cover or be enhanced by amotivation and therefore loss for willingness in participating in athletic activities. Moreover, affected motor coordination concerns motor skill learning specifically in the consolidation phase [32], which in turn signifies lower scores in perceptual ability tests (PATest) due to a rather physical etiology.

### 4.3. Visual Stimulation and Motor Skills

Motor skills can become quite complex in fast-paced activities, like soccer, since the information from the surroundings flows in rapid and multidirectional streams. In such cases, athletes rely more on their visual processing skills and, thus, hone them with training protocols, such as stroboscopic vision perturbation [33]. It is worth noting that perturbation of visual process enhances perceptual-motor tasks, in contrast to its elimination, due to increased attentional allocation and faster reaction times [34]. Despite the fact that lack of motivation can be attributed to various reasons, the impact upon sports performance and athletes' perceptual abilities remains the same. The consequences become more apparent notably in competitive periods, in which stress and its effects mentioned above act synergistically, further undermining mental clarity.

### 4.4. Age and Body Composition

Our results presented relationship between age and body composition compared to PSQI score and selected variables of PSQI questionnaire (Table 1). Adolescents claimed to be poorer sleepers according to their PSQI scores, whereas their body composition was significantly more optimal. Thus, the diminished sleep quality and quantity in this age group could be attributed to factors regardless of body composition. According to Vitale et al. [35], the ever-growing prevalence of smartphones and other electronic devices in young adults are related to sleep deprivation and/or poor sleep quality, while the use of electronic devices has been associated with less sleep at night, probably because the light produced by electronic devices may disrupt circadian rhythms by suppressing melatonin, resulting in the inability to fall asleep at a reasonable time [36]. Sleep-wake cycle is subjected to circadian rhythm which, in contrary to popular belief, differs in puberty and adulthood. Melatonin in puberty is secreted comparatively later than in adulthood [37]. Due to this social misconception, adolescents are faced with delayed sleep phase, worsening their quality and quantity [38]. The disruption of sleep-wake cycle has been shown to reserve detrimental consequences, even in adolescents, by hindering the neuroplastic capability of honing social and cognitive skills [39].

Furthermore, sleep fragmentation is responsible for the disruption of the hypothalamic-pituitary-adrenal (HPA) axis, elevating cortisol levels [40]. Steroid hormones (testosterone, cortisol/corticosterone) affect the nocturnal catabolic debt conditions and they appear to be associated with increased adipose body mass in humans, which in turn diminishes performance in exercise due to suboptimal body composition [41], besides cognitive decline because of sleep deprivation. Cortisol enhances the activity of catecholamines and, hence, the SNS activity. Hypercortisolemia enhances arousal and vigilance up to a specific level in athletes [42], whereas, in excess levels, the benefits are reversed [43]. Hence, sleep deprivation does impact not only immunity, but also neuronal hubs' organization, signifying that the immune system is mutually related to brain functions [44].

### 4.5. Limitations

In our study, there were some limitations. The nocturnal polysomnography, as a gold standard for the diagnosis of sleep disordered breathing, and the chronotype, factor that influences athletes' sleep, were not evaluated. Color blindness, which is common in men as it affects approximately one in twelve (8%) [45], was not assessed in the present study. The possibility of trouble of seeing red and green colors during PATest, leading to false lower scores, cannot be excluded. Moreover, the possible involvement of immune and pro-inflammatory markers was not assessed in the present study, as it seems to drive sleep-wake cycle based on maturation stage [46].

## 5. Conclusion

To sum up, sleep parameters, indicative of suboptimal sleep quality, were associated with lower reaction times, principally in adolescents, after exhaustive exercise. Sleep disturbances were associated with declines in perceptual ability's domains. Perceptual ability, which requires sustained attention, vigilance, and motor coordination, is often negatively affected by restricted sleep. It is suggested to inform athletes, regardless of age, about strategies of sleep hygiene, to prevent the detrimental effects of sleep deprivation in perceptual ability, athletic performance, and recovery.

## Figures and Tables

**Table 1:** Athletes' characteristics results between adolescents and adults. Continuous variables are presented as mean ± standard deviation. The relationship between hits, reaction time, and PSQI score and age group are presented as *P* value.

	Total *n* = 86	Adolescents *n* = 35	Adults *n* = 51	*P* value†	95% Cl	Hits (rep/30 s)		Reaction time (sec)		PSQI score	
						Adolescents	Adults	Adolescents	Adults	Adolescents	Adults
Age, yrs	22.7 ± 6.0	17.3 ± 0.2	26.3 ± 5.2	**<0.001**	−10.814–−7.223	0.251	0.472	0.345	0.907	**0.013**	0.181
Training age, yrs	9.3 ± 4.9	6.5 ± 2.2	11.1 ± 5.4	**<0.001**	−6.516–−2.672	0.202	0.209	0.144	0.463	0.811	0.517
Body mass, kg	73.5 ± 8.3	68.9 ± 7.9	76.5 ± 7.2	**<0.001**	−10.877–−4.336	0.275	0.629	0.417	0.255	0.462	0.359
Body fat, %	10.1 ± 3.3	9.9 ± 3.6	10.3 ± 3.1	0.601	−1.850–1.078	0.870	0.719	0.629	0.666	0.651	0.208
Body mass index, kg/m^2^	22.8 ± 1.7	21.9 ± 1.8	23.5 ± 1.3	**<0.001**	−2.239–−0.879	0.682	0.576	0.821	0.328	0.302	0.235
Muscle mass, kg	62.2 ± 7.3	57.9 ± 7.1	65.2 ± 5.7	**<0.001**	−0.326–−0.114	0.326	0.750	0.513	0.306	0.267	0.527
Δchest, cm	6.6 ± 1.9	7.0 ± 1.8	6.3 ± 1.9	0.097	−5.947–−2.010	0.109	0.655	0.137	0.473	0.864	0.455
Body surface area, m^2^	1.8 ± 0.3	1.7 ± 0.2	1.9 ± 0.2	**<0.001**	−1.287–0.861	0.491	0.596	0.674	0.283	0.707	0.331
Lean body mass, %	58.5 ± 4.9	56.2 ± 4.6	60.1 ± 4.5	**<0.001**	−10.111–−4.587	0.475	0.667	0.656	0.292	0.738	0.354
Total body water, %	64.1 ± 2.4	63.9 ± 2.6	64.2 ± 2.4	0.694	−0.130–1.532	0.479	0.859	0.579	0.802	0.236	0.281
*V*O_2resting_, ml/min^−1^/kg^−1^	6.2 ± 1.5	6.9 ± 1.7	5.9 ± 1.4	0.123	−0.143–1.182	0.810	0.060	0.727	0.115	0.976	0.273
*V*O_2anaerobic threshold,_ ml/min^−1^/kg^−1^	45.2 ± 4.2	46.4 ± 4.6	44.3 ± 3.7	**0.021**	0.321–3.901	0.686	0.709	0.474	0.693	0.595	0.149
*V*O_2max_, ml/min^−1^/kg^−1^	58.1 ± 4.9	59.7 ± 6.2	57.0 ± 3.6	**0.012**	0.617–4.801	0.365	0.871	0.519	0.453	0.931	0.088
HR_resting_, bpm^−1^	99.9 ± 16.5	100.8 ± 15.0	99.2 ± 17.6	0.663	−1.619–0.987	0.757	0.784	0.055	0.841	0.349	0.680
HR_peak_, bpm^−1^	193.9 ± 10.1	197.9 ± 8.3	191.1 ± 10.3	**0.002**	−5.650–8.837	0.734	0.863	0.680	0.709	**0.032**	0.164
ΔHR, bpm^−1^	22.9 ± 8.7	30.1 ± 11.0	31.0 ± 12.2	0.777	2.673–11.055	0.197	0.768	0.163	0.697	0.077	0.751
Duration 2^nd^ stage, min^−1^	11.7 ± 1.2	11.7 ± 1.4	11.7 ± 1.1	0.798	−0.943–11.485	0.868	0.659	0.870	0.968	0.850	0.011
Speed_peak_, km/h^−1^	17.6 ± 1.1	17.6 ± 1.3	17.7 ± 1.0	0.705	−5.833–4.372	0.607	0.995	0.695	0.746	0.844	0.016

HR = heart rate, RT = reaction time, *V*O_2_ = oxygen uptake, Δchest = circumference difference between maximal inhalation and exhalation, ΔHR = beats per minute difference between maximal effort and 1^st^ minute of recovery, † between groups, 95% Cl = 95% confidence Interval of the difference.

**Table 2:** Analysis between PSQI results and age group. Continuous variables are presented as mean ± standard deviation. The relationship between reaction time and age group is presented as *P* value.

	Total *n* = 86	Adolescents *n* = 35	Adults *n* = 51	*P*†	95% Cl	Reaction time (sec)	
						Adolescents	Adults
How long has it taken you to fall asleep each night?	13.9 ± 9.2	14.5 ± 8.6	13.6 ± 9.8	0.660	−3.167–4.971	0.061	0.477
What time have you usually gotten up in the morning?	9.2 ± 2.2	9.3 ± 1.4	9.1 ± 2.6	0.711	−0.794–1.160	0.722	0.987
How many hours of actual sleep did you get at night?	7.7 ± 1.3	7.6 ± 1.4	7.8 ± 1.3	0.645	−0.717–0.446	0.939	0.749
Cannot get to sleep within 30 minutes	0.7 ± 0.9	0.9 ± 1.0	0.6 ± 0.9	0.053	−0.005–0.811	0.168	0.127
Wake up in the middle of the night or early morning	0.9 ± 1.0	1.5 ± 1.1	0.6 ± 0.9	**<0.001**	0.377–1.242	0.908	0.713
Have to get up to use the bathroom	0.8 ± 0.9	1.1 ± 0.9	0.6 ± 0.8	**0.007**	0.152–0.938	0.106	0.621
Cannot breathe comfortably	0.1 ± 0.2	0.1 ± 0.4	0.1 ± 0.1	**0.046**	0.002–0.226	**0.014**	0.693
Cough or snore loudly	0.2 ± 0.5	0.3 ± 0.7	0.1 ± 0.2	**0.020**	0.040–0.470	0.087	0.764
Feel too cold	0.1 ± 0.3	0.1 ± 0.3	0.1 ± 0.2	0.255	−0.055–0.205	0.158	0.077
Feel too hot	0.7 ± 1.0	1.1 ± 1.0	0.4 ± 0.8	**0.001**	0.308–1.153	0.190	0.642
Have bad dreams	0.3 ± 0.6	0.4 ± 0.7	0.1 ± 0.4	**0.012**	0.073–0.566	**0.035**	0.729
Have pain	0.1 ± 0.2	0.1 ± 0.1	0.1 ± 0.2	0.794	−0.091–0.070	0.734	0.673

During the past month…							
…how often have you taken medicine to help you sleep?	0.1 ± 0.1	0.1 ± 0.1	/±/	—	−0.018–0.075	0.743	—
…how often have you had trouble staying awake while driving, eating meals, or engaging in social activity?	0.1 ± 0.4	0.3 ± 0.6	0.1 ± 0.2	**0.027**	0.023–0.373	0.494	0.830
…how much of a problem has it been for you to keep up enthusiasm to get things done?	0.3 ± 0.5	0.4 ± 0.6	0.1 ± 0.4	**0.009**	0.075–0.507	0.241	0.348
…how would you rate your sleep quality overall?	0.8 ± 0.7	0.9 ± 0.7	0.7 ± 0.6	0.219	−0.139–0.460	0.471	0.376

Questions 5–13 [scale: not during the past month (0), less than once a week (1), once or twice a week (2), three or more times a week (3)]; Questions 14–17 [scale: very good (0), fairly good (1), fairly bad (2), very bad (3)], RT = reaction time, † between groups, 95% Cl = 95% confidence Interval of the difference.

## Data Availability

The data are available on request.
